# Comparative Evaluation of Chiglitazar and Sitagliptin on the Levels of Retinol-Binding Protein 4 and Its Correlation With Insulin Resistance in Patients With Type 2 Diabetes

**DOI:** 10.3389/fendo.2022.801271

**Published:** 2022-04-25

**Authors:** Yunting Zhou, Huiying Wang, Yuming Wang, Xiaohua Xu, Fengfei Li, Junming Zhou, Ting Shan, Rong Huang, Tingting Cai, Xiaomei Liu, Xiaofei Su, Huiqin Li, Jianhua Ma

**Affiliations:** ^1^Department of Endocrinology, Nanjing First Hospital, Nanjing Medical University, Nanjing, China; ^2^Department of Cadre Gastroenterology, Jinling Hospital, Medical School of Nanjing University, Nanjing, China

**Keywords:** type 2 diabetes mellitus, chiglitazar, sitagliptin, insulin sensitivity, RBP-4

## Abstract

**Aims:**

We evaluated the efficacy and significant changes in the levels of retinol-binding protein 4 (RBP-4) and insulin resistance in patients with type 2 diabetes mellitus (T2DM) treated with chiglitazar versus sitagliptin.

**Methods:**

Eighty-one T2DM patients with haemoglobin A1c (HbA1c) level of 7.5%–10.0% were selected. Based on the study criteria, patients were randomly assigned to receive chiglitazar (32 mg), chiglitazar (48 mg), or sitagliptin (100 mg) orally for 24 weeks. Sociodemographic and anthropometric characteristics, lipid profiles, glucose profiles, and serum RBP-4 levels were determined at baseline and at the end of the therapy.

**Results:**

After treatment for 24 weeks, significant changes in fasting blood glucose (FBG), fasting insulin (Fins), 2 h-blood glucose (2h-BG), the score values of insulin resistance/insulin secretion/β cell function (HOMA-IR, HOMA-IS, and HOMA-β), triglyceride (TG), free fatty acid (FFA), high-density lipoprotein cholesterol (HDL-C), and RBP-4 levels were detected in patients with chiglitazar administration and sitagliptin administration. Changes in RBP-4 levels were positively correlated with changes in HOMA-IR and 2 h-BG in linear regression.

**Conclusions:**

Chiglitazar showed a greater improvement in parameters of diabetes than sitagliptin, and changes in serum RBP-4 levels were associated with changes in insulin-sensitizing parameters.

**Clinical Trial Registration:**

ClinicalTrials.gov, CT.gov identifier: NCT02173457.

## Introduction

Diabetes mellitus (DM) is a metabolic disease characterized by insulin resistance and systemic lipid disorder and is one of the most serious public health challenges worldwide (1). This situation in China is particularly worrisome, where nationwide representative surveys have reported that the estimated overall prevalence of diabetes increased to 10.6% (2). Almost 70% of type 2 DM (T2DM) is characterized by insulin secretion deficiency and insulin resistance with concurrent diabetic dyslipidaemia (3).

An important therapeutic method for maintaining glycaemic control is administering insulin sensitizers that stimulate peroxisome proliferator-activated receptors (PPARs) or administering inhibitors of dipeptidyl peptidase-4 (DPP-4), which are anti-hyperglycaemic agents. Chiglitazar is a PPAR agonist that has been newly identified as a nonthiazolidinedione (TZD) insulin sensitizer, and it has a balanced and moderate activation of the receptors of the PPAR-α, γ and δ isotypes (4). Previous *in vitro* and *in vivo* phase I and IIa studies have shown that chiglitazar has different activities and efficacy characteristics from TZD drugs (4, 5). Due to its higher stability and bioavailability, sitagliptin, an inhibitor of DPP-4, emerged as a member of a new class of anti-hyperglycaemic agents. It has shown beneficial effects on glycaemic control and islet mass and function, and it can reduce haemoglobin A1c (HbA1c) levels and prevent hypoglycaemia, with no relevant adverse effects (6). Recent studies demonstrated sitagliptin has the ability to improve the insulinogenic index, increase β cell neogenesis, and reduce glucagon secretion (7, 8). Because of the vast range of physiological actions promoted by incretins, sitagliptin also presents beneficial effects on prevention of diabetes serious complications such as cardiac and neuronal diseases.

Serum retinol binding protein 4 (RBP-4) belongs to a family of soluble proteins that is found in many cells as well as in extracellular compartments, and it exhibits high selectivity and affinity towards different types of vitamin A metabolites called retinoids (9, 10). Both clinical and basic studies demonstrated that serum levels of RBP-4 were positively correlated with improved insulin resistance parameters and inversely associated with islet function (11, 12). Further prospective cohort studies confirmed the predictive value of serum RBP-4 levels as an independent risk factor for prediabetes populations (13, 14). However, the efficacy of chiglitazar and sitagliptin on RBP-4 expression remains poorly understood. In the current study, we aimed to assess the comparative effect of chiglitazar and sitagliptin on the levels of RBP-4 and insulin resistance in patients with T2DM who had insufficient glycaemic control and to further investigate the significance of the changes in the levels of RBP-4, which is identified as a potential metabolic health biomarker and outcome for the evaluation of islet function.

## Patients and Methods

### Study Design and Population

This study was a randomized, double-blind phase 3 trial conducted at Nanjing First Hospital in China. Between December 2014 and May 2016, eligible patients at the Department of Endocrinology in Nanjing First Hospital who met the study criteria were randomly allocated (1:1:1) to receive chiglitazar (32 mg), chiglitazar (48 mg), or sitagliptin (100 mg) orally for 24 weeks. Under the condition that the standard deviation of each group is 1.4% and the two-sided test level is 0.05, the ratio of 1:1:1 is included. Considering power and significance level were 80% and 0% respectively, the sample size of this study was calculated and expected to be included about 84 subjects for randomized group. Finally, a total of 81 patients completed the study. The inclusion criteria were as follows: 1) Aged over 18 years and diagnosed with T2DM according to World Health Organization 1999 diagnostic criteria of T2DM; 2) Insufficient glycemic control (HbA1c 7.5-10%) in spite of strict diet and exercise therapy; 3) Body mass index (BMI) (18.5-35.0 kg/m2); 4) Willing to randomly participant in the treatment groups. The exclusion criteria were as follows: 1) T1DM; 2) Fasting blood glucose (FBG) over 13.3 mmol/L; 3) A history of insulin, traditional Chinese medicine or other antidiabetic therapies; 4) Non-controlled hypertension blood pressure (BP); 5) Triglyceride (TG) > 500 mg/dL; 6) History of vascular or unstable angina accident in the past 6 months; 7) History of drug therapies which contribute to blood glucose control such as adrenocorticosteroids, steroids and so on; 8) Impaired pancreatic, hepatic and renal function; 9) Severe tumors in the past 5 years. All patients, investigators, study site staff, and sponsors were blinded to the treatment assignment until the database was locked. Finally, after excluding those who missed baseline demographic data collection or laboratory index tests, a total of 81 participants were included in the final statistical analysis.

### Experimental Procedures

For the placebo lead-in period, participants were requested to take three chiglitazar placebo tablets and one sitagliptin placebo tablet each day for 2 weeks. For the double-blind treatment period, participants were requested to take their corresponding drugs 30 minutes after breakfast each day (the first medication could be taken after completing all the checks in the baseline period, or it could be taken 30 minutes after breakfast the next day). The participants were required to visit the local study centres every four weeks. Participants were asked to fast for more than 12 h prior to their exam. At the study centre, participants provided an initial blood sample for measuring fasting blood glucose (FBG). Participants completed an oral glucose tolerance test with blood glucose measured at 2 h (2 h-BG). During the 2 h waiting period, BP, height, and waist circumference were measured and calculated using standardized protocols. BMI was calculated as body weight divided by height squared (kg/m^2^). Blood samples were collected into test tubes in the presence of 0.1% EDTA. Collected blood samples were then centrifuged to separate the plasma within 15 min of collection and stored at −80°C for further analysis.

### Food Intake

During the treatment period, participants were instructed to maintain physical activity according to their doctors’ personalized instructions. Patients were instructed to have breakfast, lunch, and dinner at 7: 00 am, 11: 00 am, and 5: 00 pm, respectively. Patients were educated to keep in the consistent diet and exercise habits and asked to measure their FBG levels at least once a week using a blood glucose metre during the study period.

### Serum Profiles Measurements

HbA1c was tested using a high-performance liquid chromatography assay (Bio–Rad Laboratories, Inc. CA, USA). Serum biochemical parameters, including FBG, alanine aminotransferase (ALT), aspartate aminotransferase (AST), TC, TG, FFA, HDL-C, and LDL-C, were measured with routine laboratory methods using a HITACHI 7600 device (HITACHI, Tokyo, Japan). Levels of insulin were measured with a radioimmunoassay kit. Levels of RBP-4 were determined by a latex immunoturbidimetric kit (Jingmei, Inc. China). The estimated glomerular filtration rate (eGFR) was calculated using the Modification of Diet in renal disease equation: eGFR (mL/min/1.73 m^2^) =186 × (SCr/88.4)-1.154× (age)-0.203× (0.742 if female). The homeostasis model assessment for insulin resistance (HOMA-IR), β cell function (HOMA-β), and insulin secretion (HOMA-IS) were assessed through previously published procedures (15).

### Statistical Analysis

Continuous variables in this study are described as the means ± SEM or median (25th - 75th percentile). Categorical data are presented as percentages (%). One-way variance (ANOVA) for continuous variables and/or χ2 test for categorical variables was used to compare the characteristics of participants in the different treatment groups. A nonparametric test was used when the data distribution was skewed. Correlations were determined by Spearman correlation coefficients. P <0 .05 was considered statistically significant. All statistical analyses were performed using SPSS 22.0 (SPSS Inc., USA) and GraphPad Prism 6.0 (GraphPad Inc., USA).

## Results

### Baseline Characteristics

**Figure 1:**
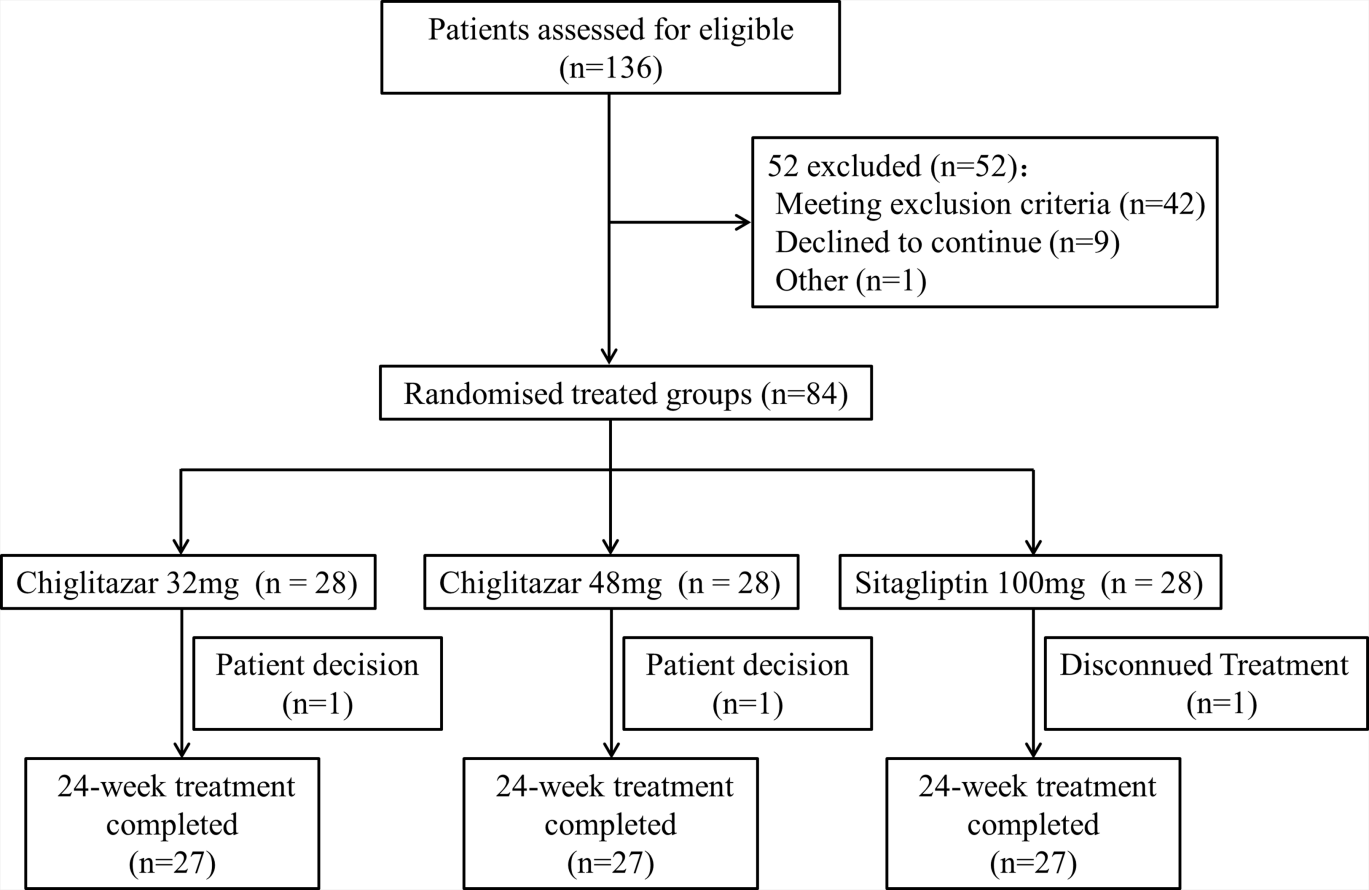
Flowchart of the trial study.

A total of 81 T2DM patients who completed the 24-week treatment were enrolled (males: females=50:31), because 52 patients did not meet the inclusion criteria and 3 patients reconsidered their decision or discontinued their treatment (**Figure 1**). No significant differences in terms of age, history of diabetes, BP, heart rate (HR), body weight and BMI were observed among the three groups of study participants. Additionally, for hepatic and renal function-related tests, there were no notable effects or differences in serum ALT, AST, creatinine (Cr) or GFR among the groups. Similarly, no statistically significant differences were found in lipid metabolism parameters (TC, TG, FFA, LDL-C, and HDL-C), glucose metabolism parameters (FBG, 2h-PBG, HbA1c), and serum RBP-4 levels at baseline among participants from these three study groups (**Table 1**).

**Table 1:** Baseline characteristics of study patients.

	Sitagliptin100mg(N=27)	Chiglitazar32mg(N=27)	Chiglitazar48mg(N=27)	P
Sex, men, female (n, %)	15/12(55.6/44.4)	20/7(74.0/26.0)	18/19(66.7/33.3)	0.571
Age (years)	53.52 ± 9.90	52.74 ± 9.10	55.11 ± 7.53	0.607
History of diabetes (years)	1.45 ± 0.30	1.59 ± 0.23	1.48 ± 0.21	0.530
HR (bpm)	77.44 ± 1.87	75.88 ± 1.73	76.66 ± 2.05	0.845
Waist (cm)	87.83 ± 1.35	89.29 ± 1.80	88.25 ± 1.91	0.824
Weight (kg)	67.51 ± 1.59	73.27 ± 2.00	68.68 ± 2.24	0.824
BMI (kg/m2)	25.43 ± 0.48	26.94 ± 0.73	25.28 ± 0.53	0.659
Systolic BP (mmHg)	128.07 ± 2.61	129.18 ± 3.45	126.13 ± 2.46	0.750
Diastolic BP (mmHg)	76..85 ± 1.67	76.48 ± 2.33	73.14 ± 2.62	0.440
ALT (U/L)	25.78 ± 1.96	30.74 ± 3.85	25.30 ± 1.95	0.091
AST (U/L)	23.56 ± 1.83	25.33 ± 3.26	21.22 ± 1.53	0.243
Cr (μmol/L)	72.56 ± 3.31	75.74 ± 3.47	72.59 ± 2.69	0.719
GFR(mL/min/1.73 m2)	100.73 ± 6.97	115.84 ± 14.87	92.37 ± 4.89	0.464
TC (mmol/L)	4.89 ± 0.14	4.75 ± 0.17	4.98 ± 0.19	0.614
TG (mmol/L)	2.00 ± 0.24	2.22 ± 0.33	1.88 ± 0.25	0.683
FFA (mmol/L)	0.52 ± 0.04	0.51 ± 0.03	0.51 ± 0.04	0.898
LDL-C (mmol/L)	2.90 ± 0.11	2.60 ± 0.16	2.97 ± 0.16	0.177
HDL-C (mmol/L)	1.40 ± 0.30	1.08 ± 0.46	1.18 ± 0.05	0.444
FBG (mmol/L)	8.98 ± 0.43	8.70 ± 0.29	9.61 ± 0.48	0.268
2 h-PBG (mmol/L)	15.70 ± 0.72	14.57 ± 0.58	15.83 ± 0.68	0.352
HbA1c (%)	8.21 ± 0.15	8.30 ± 0.16	8.33 ± 0.14	0.835
RBP-4 (mg/L)	41.91±1.71	42.37 ± 1.43	43.32 ± 1.74	0.830

Data were shown as mean (SEM) or n (%), BMI, body mass index; SBP, systolic blood pressure; ALT, alanine aminotransferase; AST, aspartate transaminase; eGFR, estimated glomerular filtration rate; TC, total cholesterol; TG, triglycerides; HDL-C, high-density lipoprotein cholesterol; LDL-C, low-density lipoprotein cholesterol; FBG, fasting blood glucose; PBG, postprandial blood glucose; HbA1c, glycosylated hemoglobin; RBP-4, retinol-binding protein 4.

### Glycaemic Metabolism Profiles

**Figure 2:**
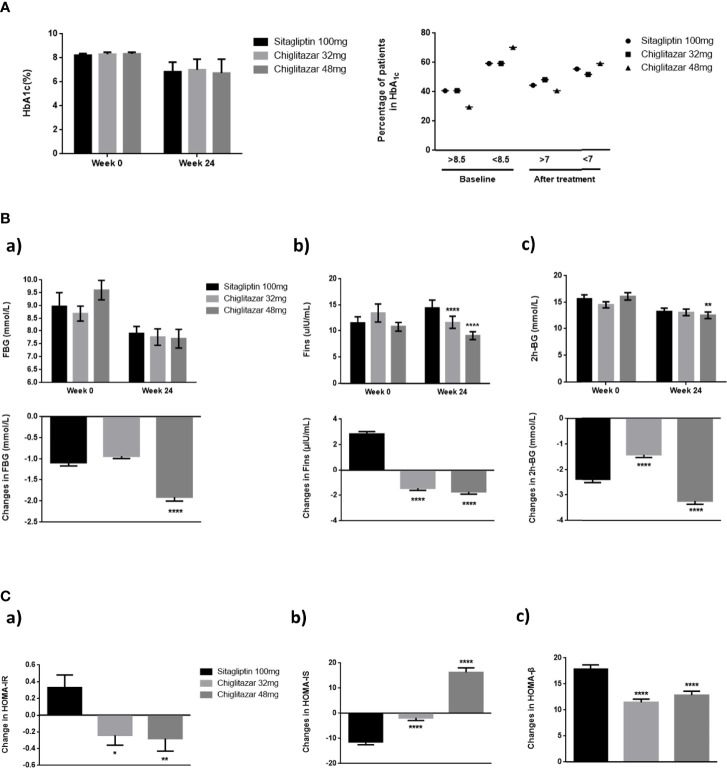
Changes of glycaemic control parameters in groups. **(A)** Mean HbA1c of groups from baseline to week 24, and percentage of patients who arrived HbA1c < 7%. **(B)** Mean FBG, Fins, and 2 h-BG of groups from baseline to week 24. **(C)** Changes of HOMA-IR, HOMA-IS, and HOMA-β from baseline to week 24. *P < 0.05, **P < 0.01, ****P < 0.0001. Error bars show standard error of the mean (SEM).

As shown in **Figure 2**, there were no significant reductions in HbA1c levels from baseline to week 24 in the full analysis population, with values of -1.29%, -1.60%, and -1.36% for chiglitazar 32 mg, chiglitazar 48 mg, and sitagliptin 100 mg, respectively (**Figure 2A**). Similarly, 48 mg chiglitazar showed a greater reduction than 100 mg sitagliptin in FBG levels, fasting insulin (Fins), and 2 h-BG at week 24, with values of -1.91 ± 0.48 vs. -1.08 ± 0.41 (P<0.0001) and -1.72 ± 0.99 vs. -2.84 ± 1.03 (P<0.0001) and -3.23 ± 0.67 vs. -1.42 ± 0.60 (P<0.0001), respectively (**Figure 2B**). Differences in the insulin sensitivity analyses were similar among the treatments. The HOMA-IR values in the chiglitazar at 48 mg group were significantly lower than those in chiglitazar at 32 mg and sitagliptin at 100 mg groups, with change values of -0.28 ± 0.15, -0.24 ± 0.12, and 0.33 ± 0.15, respectively. HOMA-β in the chiglitazar at 32 mg and chiglitazar at 48 mg groups was significantly lower over the administration period than that in the sitagliptin at 100 mg group. The HOMA-IS in the chiglitazar groups was significantly higher over the administration period than that in the sitagliptin at 100 mg group (**Figure 2C**).

### Lipid Metabolism and Serum RBP-4 Profiles

**Figure 3:**
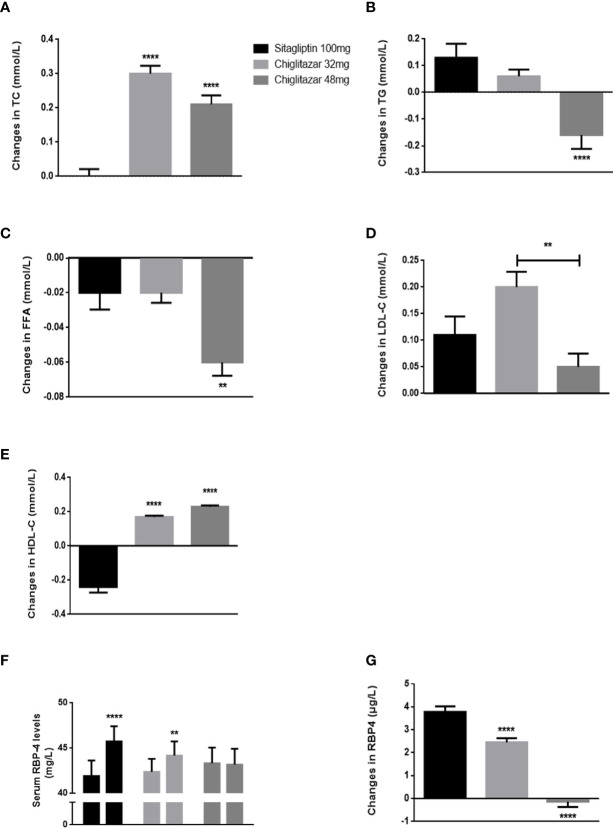
Changes of lipid metabolism parameters and serum RBP-4 levels in groups. **(A–E)** changes of TG, FFA, TC, LDL-C, and HDL-C shown from baseline to week 24. **(F)** mean RBP-4 levels of groups from baseline to week 24. **(G)** changes of RBP-4 levels shown from baseline to week 24. **P < 0.01, ****P < 0.0001. Error bars show standard error of the mean (SEM).

Changes in lipid metabolism parameters over treatment time from the central laboratory analysis are summarized in **Figure 3**. Our data showed that for the subjects, the levels of TG were changed at the end of the trial compared to baseline among the three groups. A greater reduction in TG was observed in subjects in the chiglitazar at 48 mg group than those in the chiglitazar at 32 mg group, while TG was increased in the sitagliptin group (**Figure 3B**). FFA levels from participants in the chiglitazar at 48 mg group were significantly changed at the end of trial compared to baseline. The change value of this group was -0.06 ± 0.04, and the change value of the chiglitazar 32 mg group was -0.02 ± 0.05 (**Figure 3C**). A slight reduction in blood LDL-C was noted in the chiglitazar at 48 mg group compared with the sitagliptin group (**Figure 3D**). The significant reduction in body weight and BMI (data not shown) were also detected in the chiglitazar at 48 mg group compared with the sitagliptin group, whose tendency were consistent with the changes of TG, FFA and LDL-C levels. Compared with sitagliptin 100 mg, chiglitazar at both doses showed substantial increases in TC and HDL-C levels (**Figures 3A, E**). Sitagliptin 100 mg and chiglitazar 32 mg showed significant increase from baseline to week 24 with values of 0.9% and 0.42% respectively. Simultaneously, chiglitazar 48 mg showed a slight reduction in RBP-4 levels at weeks 24 (**Figure 3F**). Further analysis showed the three treatment groups showed declining the accumulation of RBP-4 levels at week 24, with change values of 3.79 ± 1.23, 2.45 ± 0.96, and -0.14 ± 1.19 for the sitagliptin at 100 mg, chiglitazar at 32 mg, and chiglitazar at 48 mg groups, respectively (**Figure 3G**).

### Linear Correlation Between Changes in Serum RBP-4 Levels and Insulin Sensitivity Assessments

**Figure 4:**
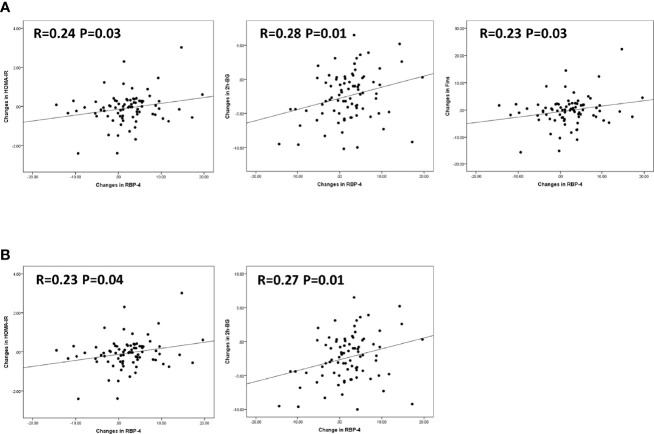
Correlation between changes in serum RBP-4 levels and various insulin sensitivity parameters. **(A)** Changes in HOMA-IR, 2h-BG, and Fins showed positive linear correlations with changes in RBP-4 levels in linear regression. **(B)** With adjustment for gender, age, and BMI, changes in HOMA-IR, and 2h-BG showed positive linear correlations with changes in RBP-4 levels in linear regression.

Finally, we wanted to validate that the observed changes in serum RBP-4 levels following chiglitazar 32 mg, chiglitazar 48 mg, and sitagliptin 100 mg treatment could contribute to the improved insulin resistance observed in these patients. A correlation analysis between the changes in serum RBP-4 levels and glycaemic control and insulin resistance parameters, including HOMA-IR, HOMA-β, and HOMA-IS scores and FBG, Fins, and 2 h-BG levels, was performed for all participants. Significant positive linear correlations were indeed observed between the changes in serum RBP-4 levels and HOMA-IR (R=0.24, P<0.05), Fins (R=0.23, P<0.05), and 2 h-BG (R=0.28, P<0.05) (**Figure 4A**). A similar correlation was not detected in HOMA-β, HOMA-IS, or FBG. After further adjusting for gender, age, and BMI, serum RBP-4 levels correlated positively with HOMA-IR (R=0.23) and 2 h-BG (R=0.27) (p < 0.05) (**Figure 4B**).

## Discussion

Our present study demonstrated that chiglitazar treatment could significantly improve glycaemic control parameters (such as FBG, Fins, 2 h-BG, HOMA-IR, HOMA-IS, and HOMA-β) and tended to have a greater reduction in lipid metabolism parameters (such as TG and FFA, rather than TC, LDL-C, and HDL-C) than sitagliptin in T2DM patients. The results also showed for the first time that both doses of chiglitazar had a greater efficacy on the downregulation of serum RBP-4 levels than sitagliptin, which correlated with insulin sensitivity parameters, including HOMA-IR, Fins, and 2 h-BG.

Chiglitazar, similar to other known PPAR panagonists such as muraglitazar, naveglitazar, tesaglitazar, and KRP-297, is a configuration-restricted non-TZD PPAR panagonist with AC50 values of 1.2, 0.08, and 1.7 μM in CV-1 cells for PPARα, PPARγ, and PPARδ, respectively, and it is currently in phase III clinical trials in China (16). The glycaemic efficacy of the first oral sitagliptin had undergone randomized controlled clinical trials along with the available antidiabetic agents in China (17, 18). In terms of the underlying mechanism of anti-diabetes treatment, chiglitazar is considered to be similar to sitagliptin, rather than metformin. Given the consideration of the relatively neutral effects of sitagliptin on severe hypoglycaemia and body weight loss, we chose sitagliptin as a suitable active comparator in our study. After 24 weeks of drug administration in our study, the levels of FBG and Fins were significantly lowered among the three groups, and a potentially greater reduction in 2 h-BG was observed in the chiglitazar at 48 mg group than the sitagliptin at 100 mg group. Along with improved glycaemic control, changes in HOMA-IR, HOMA-IS, and HOMA-β scores also indicated a greater effect on insulin sensitivity parameters in the 48 mg chiglitazar group. Our results were quite consistent with previous preclinical animal studies showing that chiglitazar sodium demonstrated comparable antidiabetic effects in KK-Ay and db/db diabetic mouse models (5). PPAR panagonists have also been confirmed to significantly reduce abnormally elevated blood glucose levels without causing hypoglycaemia, improve insulin resistance, and moderate impairment of insulin secretion (19, 20). Therefore, it was reasonable to consider that the observed glycaemic effects in this study might be related to its protective ability as an insulin sensitizer.

Our results also showed that 48 mg chiglitazar decreased TGs and FFAs over time, and the mean changes from baseline to the end point were significantly greater with chiglitazar than with sitagliptin. These treatments can significantly reduce the accumulation of TG in the liver caused by a high-fat diet, inhibit the formation of visceral fat, and increase insulin sensitivity of tissues such as liver, muscle, and fat (21). The molecular mechanisms of the biological responses of PPARs can be switched on or off by binding to small lipophilic compounds, which results in either transactivation or transrepression of the target genes. Chiglitazar significantly upregulated the expression of PPARα and/or PPARδ downstream genes involved in the key processes of lipid metabolism and thermogenesis (22). In the DNA-dependent process of transactivation, the heterodimerization of PPARγ with retinoid X receptor (RXR) and the recognition of DNA response elements, peroxisome proliferator response elements (PPREs) were produced in the promoter regions of the target genes. Conformational changes in PPARs result in the recruitment of cofactors and co-activators. These co-activators interact with nuclear receptors in a ligand-dependent way and influence a set of transcribed genes (23, 24). Furthermore, some newly identified non-TZD-type agonists can partially stimulate PPARγ-mediated downstream signaling *via* modulating its phosphorylation status at the ser273 site caused by interaction of ligand with previously unrecognized β-sheet domain (25). Therefore, activation can enhance reverse cholesterol transport from peripheral tissues, thereby increasing the overall plasma cholesterol content in both HDL-C and LDL-C lipoprotein particles (26). These results preliminarily verify that compared with sitagliptin, chiglitazar sodium has a certain greater regulatory effect on blood lipids, which were observed for TG and FFA but not TC and HDL-C. These findings should be further confirmed with larger sample sizes and better control of the analysis of confounding factors in the future.

Given the close association between elevated RBP-4 levels and glycaemic metabolism and insulin resistance in T2DM patients (27), longitudinal studies are needed to investigate how these changes in RBP-4 levels occur, especially in T2DM patients predisposed to these conditions. A study by Matthews et al. (14) found that rosiglitazone reduces the long-term incidence of diabetes by delaying the underlying disease process and not reversing it. In a similar pattern, although our study detected markedly down-regulated RBP-4 expression, there was a greater reduction of RBP-4 expression by chiglitazar at both doses compared with sitagliptin. Additionally, we found significant positive linear correlations between RBP-4 levels and HOMA-IR and 2 h-BG, which demonstrate that the changes in RBP-4 levels likely contributed to the improved insulin sensitivity in T2DM patients in these treatment groups. Considering that no correlation between RBP-4 and HOMA-β, HOMA-IS, and FBG was detected, this discrepancy may be attributed to multiple factors. Limitations should also be addressed. Firstly, upon glucose stimulation, the indices are more sensitive for probing inadequate β-cell responsiveness. HOMA-β and HOMA-IS are estimates that only consider fasting/basal plasma glucose and insulin concentrations and have a relatively low precision in predicting β-cell function. Secondly, in previous studies, the correlation between RBP-4 and β-cell function was inferred by the cross-sectional design. Thirdly, the small sample size in our study may be a limiting factor. The sample size of this study was limited to a single racial centre, which should be expanded to be more convincing. Despite the observed greater effect on glycaemic control in the chiglitazar at 48 mg and chiglitazar at 32 mg groups, the correlation couldn’t be detected in each group at the end of trial in our cohort studies. Furthermore, the period of this study was relatively short, which is insufficiently to answer persistent effects of chiglitazar on the glycaemic control process of the same individual. Therefore, our current study provides the first evidence to indicate that chiglitazar yields better treatment outcomes than sitagliptin in terms of glycaemic control, possibly through inhibiting the upregulation of RBP-4 levels. However, further large-scale prospective cohort studies are needed to verify the dynamic association between RBP-4 and β-cell function in the course of T2DM.

## Conclusion

In this context, our current study demonstrates that chiglitazar, especially administrated of chiglitazar at 48 mg, showed a larger improvement in glycaemic control parameters, insulin sensitivity parameters and lipid metabolism parameters compared with sitagliptin. Meanwhile, inhibiting the upregulation of RBP-4 levels may contribute to the great antidiabetic effect of chiglitazar administration. This study should provide insight into the glycaemic control benefits of chiglitazar and suggests that RBP-4 may be a potential metabolic health biomarker for the evaluation of T2DM and a therapeutic target for T2DM patients.

## Data Availability Statement

The raw data supporting the conclusions of this article will be made available by the authors, without undue reservation.

## Ethics Statement

The study protocol was approved by the Institutional Ethics Committee of Nanjing First Hospital, Nanjing Medical University (No. 2012L02694), and was in accordance with the Helsinki Declaration of 1964, as revised in 2013. All patient provided written informed consent forms to participate in the study. The patients/participants provided their written informed consent to participate in this study.

## Author Contributions

YZ, HW and YW analyzed data and wrote the manuscript. XX, FL, JZ, TS, TC, and XL performed the experiments and collected samples. XS and RH analyzed data. XS modified the manuscript. HL and JM conceived, designed and directed the study. All authors contributed to the article and approved the submitted version.

## Funding

This study was partly supported by the National Key R&D Program of China (No. 2018YFC1314103), the National Natural Science Foundation of China (No. 81870563), the Chinese National and Provincial Major Project for New Drug Innovation (Provincial: 2011A080501010; National: 2008ZX09101-002, 2013ZX09401301) and Shenzhen Municipal Major Project (2010-1746), the Xinghuo Talent Program of Nanjing First Hospital (To YZ), and Jiangsu Innovative and Entrepreneurial Talent Programme (No.JSSCBS20211546, To YZ).

## Conflict of Interest

The authors declare that the research was conducted in the absence of any commercial or financial relationships that could be construed as a potential conflict of interest.

## Publisher’s Note

All claims expressed in this article are solely those of the authors and do not necessarily represent those of their affiliated organizations, or those of the publisher, the editors and the reviewers. Any product that may be evaluated in this article, or claim that may be made by its manufacturer, is not guaranteed or endorsed by the publisher.
